# A Knockout of the *OsGAPDHC6* Gene Encoding a Cytosolic Glyceraldehyde-3-Phosphate Dehydrogenase Reacts Sensitively to Abiotic Stress in Rice

**DOI:** 10.3390/genes16040436

**Published:** 2025-04-06

**Authors:** Jin-Young Kim, Ye-Ji Lee, Hye-Mi Lee, Yoo-Seob Jung, Jiyun Go, Hyo-Ju Lee, Ki-Sun Nam, Jong-Hee Kim, Kwon-Kyoo Kang, Yu-Jin Jung

**Affiliations:** 1 Division of Horticultural Biotechnology, Hankyong National University, Anseong 17579, Republic of Korea; zino@hknu.ac.kr (J.-Y.K.); lyj7776@naver.com (Y.-J.L.); abc_772@naver.com (H.-M.L.); joseph3688@daum.net (Y.-S.J.); ju950114@naver.com (H.-J.L.); ksnam@daum.net (K.-S.N.); jonghee014@hknu.ac.kr (J.-H.K.); 2 Department of Bio-Environmental Chemistry, College of Agriculture and Life Sciences, Chungnam National University, Daejeon 34134, Republic of Korea; jy.go2369@gmail.com; 3 Institute of Genetic Engineering, Hankyong National University, Anseong 17579, Republic of Korea

**Keywords:** *GAPDHC6* gene, CRISPR/Cas9, knockout (KO) lines, salt stress, gene expression

## Abstract

Background/Objectives: The glyceraldehyde-3-phosphate dehydrogenase (GAPDH) enzyme, encoded by *OsGAPDHC6*, plays a crucial role in glycolysis while participating in various physiological and stress response pathways. Methods: In this study, the expression levels of the *OsGAPDHC1* and *OsGAPDHC6* genes were investigated over time by treating various abiotic stresses (ABA, PEG, NaCl, heat, and cold) in rice seedlings. Results: As a result, the expression levels of both genes in the ABA-treated group increased continuously for 0–6 h and then de-creased sharply from 12 h onwards. The mutational induction of the *GAPDHC6* gene by the CRISPR/Cas9 system generated a stop codon through a 1 bp insertion into protein production. The knockout (KO) lines showed differences in seed length, seed width, and seed thickness compared to wild-type (WT) varieties. In addition, KO lines showed a lower germination rate, germination ability, and germination index of seeds under salt treatment compared to WT, and leaf damage due to 3,3′-diaminobenzidine (DAB) staining was very high due to malondialdehyde (MDA) accumulation. The KO line was lower regarding the expression level of stress-related genes compared to WT. Conclusions: Therefore, the *OsGAPDHC6* gene is evaluated as a gene that can increase salt resistance in rice as it actively responds to salt stress in the early stages of growth, occurring from seed germination to just before the tilling stage.

## 1. Introduction

Abiotic stresses such as salt, cold, heavy metals, drought, and heat negatively affect growth and development in all crops, significantly reducing yields. Exposure to various abiotic stresses during crop growth causes disturbances in the plant’s metabolic processes, disrupting the plant′s morphology and physiological mechanisms, leading to lower crop productivity [1]. Several researchers have shown that abiotic stress affects three important plant stages: the pre-anthesis phase, which occurs before the flowering stage; the terminal phase, which occurs after the flowering stage; and the period of vegetative development [2]. Temperature plays a decisive role in the growth, development, and yield of plants [3]. Plants inhibit cell elongation by interrupting water flow from the xylem to adjacent kidney cells due to drought stress caused by high temperatures. Also, drought stress decreases in leaf area, plant height, and crop development due to decreased cell elongation, low mitosis, and diastolic disorders [4]. Cold stress reduces plant development and crop efficiency, resulting in yield loss. When crops are subjected to cold stress, cells and tissues become dehydrated and cause the water crystallization of cells. Therefore, at low temperatures, electrolyte leakage and water stress increase due to moisture-active pore obstruction, reduced membrane conductivity, and increased moisture viscosity, which slows down metabolism and increases oxidative stress, consuming energy and releasing free radicals [5,6]. Strigolactone, which has recently been in the spotlight, is a plant hormone involved in plant growth and development, and studies are underway on its role in plant adaptation to various abiotic stresses [7]. However, more information is still required to understand its roles in plant adaptation towards different abiotic stresses. Therefore, the metabolism of cells undergoing abiotic stress is impaired by changes in respiratory rate, and abnormal metabolites are produced by abnormal anaerobic respiration, depending on the intensity of the stress. Cell damage and metabolic changes result in reduced plant development, abnormal fruit ripening, internal discoloration (vascular browning), increased susceptibility to spoilage, and even plant mortality [8]. Salinity is one of the major abiotic stressors causing water stress in plants. This situation makes it difficult for roots to absorb water from the soil, and high-salt conditions reduce soil moisture potential. Salinity usually occurs in semi-arid and dry areas where plants experience higher rates of evaporation and evaporation throughout the year than precipitation. High salt concentrations can lead to ion toxicity and osmotic stress. It has been reported that under normal conditions, plant cells experience a higher level of osmotic stress compared to soil, and this high osmotic pressure is used to collect water and essential nutrients from soil and store them in root cells. However, for salt stress, the osmotic solution of soil exceeds the osmotic pressure of plant cells. This is because the presence of salt in the soil limits the plant′s ability to absorb water and minerals (Ca^2+^ and K^+^). In addition, the migration of Cl^−^ and Na^+^ ions within plant cells causes damage to cell membranes and cytoplasmic metabolism. Salinity negatively affects cytoplasmic metabolism, cell development, and membrane function, and also increases ROS production. All changes affect the quality and quantity of crop production. These effects include changes to seed germination, growth impairment, and damage at the plant developmental stage due to the combined effects of greater osmotic potential and specific ion toxicity [9]. 

Rice (*Oryza sativa* L.) is a globally important food crop and is a salt-sensitive crop whose grain yield decreases by more than 50% due to salt stress [10,11,12,13,14]. The exact mechanism of salt stress in rice is not yet known, but in *Zea mays*, it is known that the excessive accumulation of salt ions (Na^+^ and Cl^−^) in leaves causes damage [15]. Glyceraldehyde-3-phosphate dehydrogenase (GAPDH) is a key enzyme in the glycolytic and gluconeogenic sugar metabolic pathways and plays a central role in cellular carbon metabolism [16]. GAPDH, which encodes glyceraldehyde-3-phosphate dehydrogenase, has been commonly used as a protein model to analyze protein structures and enzymatic mechanisms, and is employed as a housekeeping gene for the relative quantification of gene expression. However, it has recently been shown to also be involved in diverse processes in mammals, such as membrane fusion, microtubule bundling, nuclear RNA export, DNA repair, phosphatase activity, transcriptional regulation, signaling cascades, and cell death [17,18,19]. In higher plants, there are three isoforms of phosphorylated GAPDH that exist in specific locations and have separate nuclei—chloroplastic glyceraldehyde 3-phosphate dehydrogenase (GAPDHA/B), plastid glyceraldehyde 3-phosphate dehydrogenase (GAPCp), cytosolic glyceraldehyde 3-phosphate dehydrogenase (GAPDHC)—and non-phosphorylated GAPDH. It is categorized as glyceraldehyde 3-phosphate dehydrogenase (NP-GAPDH) [12]. Early studies on the function and role of plant *GAPDH* genes were related to chloroplast isoform identification and photosynthesis [20,21]. Recently, research on stress response has been conducted in model crops such as *Arabidopsis* and corn. In *Arabidopsis*, *AtGAPC-1* directly reacts with hydrogen peroxide (H_2_O_2_) to mediate reactive oxygen species (ROS) signaling [22], and *GAPC-1* has been shown to be essential for the maintenance of cellular ATP levels, carbohydrate metabolism, and normal fertility in *Arabidopsis* [23]. Additionally, *GAPDHC* is upregulated in response to various stresses, such as anaerobic stress in maize (*Z. mays*) and soybean (*Glycine max*) [24,25], heat, anaerobic stress, or increased sucrose levels in *Arabidopsis* [26,27,28], and dehydration or ABA in *Craterostigma plantagineum* [29]. Recently, studies on the function and role of the *GAPDH* gene have been actively conducted in rice, a monocotyledonous model crop. *GAPDHC* in rice plays an important role in plant growth, development, and stress response [21,30], and the overexpression of the *OsGAPC3* gene can improve salinity tolerance in rice [16]. In a previous study, eight *OsGAPDH* genes were divided into four subgroups (I, cytosolic GAPDH; II, non-phosphorylated GAPDH; III, chloroplastic GAPDHB subunit; IV, chloroplastic GAPDHA subunit), and a knockout (KO) of *OsGAPDHC7*, which showed high expression levels in each rice organ, confirmed that *OsGAPDHC7* could affect energy metabolism and amino acid accumulation [31]. The functions and roles of other *GAPDH* genes in rice have not yet been revealed and need to be studied more systematically and clearly. In this study, we isolated genes encoding putative GAPC proteins in the rice genome and conducted gene expression profiling in various organs and leaf tissues exposed to various abiotic stresses. Additionally, we selected the *OsGAPDHC6* gene to generate knockout mutant lines using the CRISPR/Cas9 system. The knockout lines of the *OsGAPDHC6* gene exhibited a heightened sensitivity to salt stress compared to wild-type plants. Our results suggest that *OsGAPDHC6* plays an important role in elucidating the molecular mechanisms of salt stress through its relationship with H_2_O_2_ levels.

## 2. Materials and Methods

### 2.1. Plant Material

Dongjin (*O. sativa* L. ssp. japonica) was used for transgenic plant development and as a control. Seedlings were transplanted at 30 × 15 cm spacing in the greenhouse facility of Hankyung National University and grown. The expression levels of *OsGAPDHC1* and *OsGAPDHC6* were investigated in the seeds of the Dongjin cultivar and in the roots, leaves, and stems 3 weeks after sowing. Abiotic stresses, including 100 μM ABA, 20% PEG6000 drought, 200 mM NaCl, 48 °C heating, and 4 °C cold, were transferred to MS liquid medium and treated for 0, 3, 6, 12, and 24 h, respectively. For all samples obtained from the abiotic stress treatment, the expression patterns of the *OsGAPDHC1* and *OsGAPDHC6* genes were monitored. All experiments included three biological replicates. 

### 2.2. Phylogenetic Analysis

Phylogenetic analysis was performed based on the estimated evolutionary divergence between *OsGAPDH* genes in rice. All information was retrieved using databases such as RAP-DB, NCBI (https://blast.ncbi.nlm.nih.gov/Blast.cgi, accessed on 12 August 2022), and Gramene. Multiple alignment analysis of predicted full-length protein sequences was performed using CLUSTALX (https://www.genome.jp/tools-bin/clustalw, accessed on 12 August 2022). Phylogenetic tree construction was performed using MEGA 4.0 by the neighbor-joining (NJ) method [32].

### 2.3. Gene Editing by CRISPR/Cas9 System

*OsGAPDHC1* and *OsGAPDHC6* genomic DNA sequences were obtained from the NCBI database (https://blast.ncbi.nlm.nih.gov, accessed on 12 August 2022). Target sites were designed in the exon regions of *OsGAPDHC1* and *OsGAPDHC6*, respectively, adjacent to the protospacer-adjacent motif (PAM) using the CRISPR RGEN tool developed at Hanyang University (http://www.rgenome.net/, accessed 2 September 2022) [33] (Supplementary Figure S2, Supplementary Table S1). The four selected sgRNAs were synthesized by Bioneer Co., Ltd. (Daejeon, Republic of Korea), and the annealed oligonucleotide pairs were cloned into the pBOsC plant transformation vector via *Aar* I digestion. The recombinant vector was transformed into rice embryogenic callus using *Agrobacterium* tumefaciens strain EHA105 as previously described [34]. All transgenic callus lines were subcultured several times and maintained on the 2N6 medium (Supplementary Figure S2) as previously described [35], and the selection of transgenic plants was performed in a medium containing 6 mg/L phosphinothricin and 400 mg/L carbenicillin. Regenerated plants were transferred to pots in the greenhouse.

### 2.4. Detection of Mutation Type

Total DNA extraction was performed by sampling 100 mg of leaves and using the DNA Quick Plant Kit (Inclone, Jeonju, Republic of Korea). The selection of T-DNA insertion transgenic plants was performed using bar gene-specific primers (Bar-F 5′-CGTCAACCACTACATCGAGA-3′) and (Bar-R 5′-AAGTCCAGCTGCCAGAAA-3′), and PCR conditions were arranged according to a previously reported method [31]. To identify target site mutations, PCR reactions were applied to MiniSeq paired-end read sequencing (Illumina, San Diego, CA, USA) and analyzed using Cas-Analyzer (http://www.rgenome.net/cas-analyzer/#!, accessed 6 April 2023) [36,37].

### 2.5. ABA and Salt Stress Sensitivity Assay in Transgenic Rice Plants

For seed germination assays, 15 seeds of each transgenic rice line (T_1_) and the WT control were sterilized and submerged in a solution of water, 200 mM NaCl, and 100 μM ABA in the dark at 28 °C. The solution was replaced every 2 days to maintain the concentrations of NaCl and ABA and the amount of distilled water, respectively. The germinated seeds were measured daily, and embryo emergence was used as an indicator of germination. The germination percentage, germination ability, and germination index were measured as previously reported [38]. The root length and shoot length of the seedlings were measured on the 10th day. 

### 2.6. DAB Stainning ROS Determination of H_2_O_2_ Content in Rice Leaves

To measure tissue H_2_O_2_ levels and visualize the extent of damage due to heat stress, leaf disks were soaked in 3,3′-diaminobenzidine (DAB) solution (1 mg/mL, pH 3.8) at 25 °C for 24 h under continuous illumination [39]. Monitoring was performed at 0, 3, 12, and 24 h. The analysis of H_2_O_2_, proline, and malondialdehyde (MDA) contents was performed according to previously reported methods [40]. Chlorophyll was extracted from plant tissues using a 2:3 (*v*/*v*) acetone/hexane mixture, and absorbance was measured at 663 and 645 nm using a spectrophotometer [41].

### 2.7. qRT-PCR Analysis

Total RNA was isolated from rice plant tissues using the RNeasy plant mini kit (Qiagen, Seoul, Republic of Korea, www.qiagen.com), and reverse transcription was performed using a kit provided by GenDEPOT (Houston, TX, USA). Quantitative RT-PCR was performed using the AccuPower^®^ RT-PCR PreMix & Master Mix kit provided by Bioneer (Daejeon, Republic of Korea). The actin gene was used as an internal control for expression analysis performed using the Bio-Rad real-time fluorescence quantitative PCR instrument. Relative gene expression was calculated using the following formula: relative expression = 2^−ΔΔCT^. Primer sequences for RT-PCR analysis are listed in Supplementary Table S3.

### 2.8. Statistical Analysis

Significant differences between mean values (*p* < 0.05) were evaluated using one-way ANOVA followed by Bonferroni’s test. Statistical analyses were performed using R statistical software (version 4.4.3).

## 3. Results

### 3.1. Structural and Expression Analysis of OsGAPDHC01 and OsGAPDHC6 Genes

**Figure 1:**
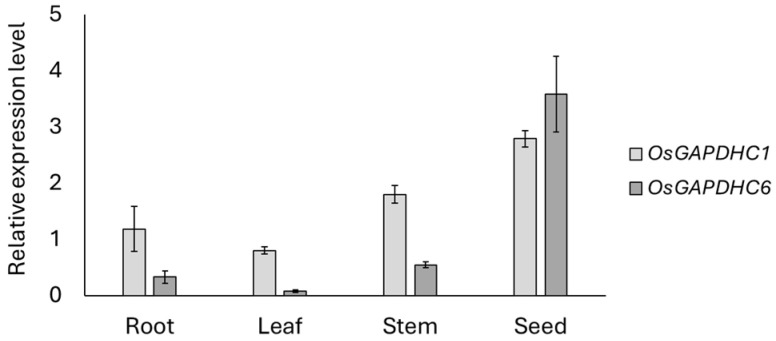
Expression levels of *OsGAPDHC1* and *OsGAPDHC6* in rice organs from seed germination to first tillering using qRT-PCR. Error bars represent standard deviation calculated from three repetitions (mean ± SD, *n* = 3).

The *OsGAPDHC1* (LOC_Os02g07490) and *OsGAPDHC6* (LOC_Os06g45590) used in this study belonged to the cytosolic GAPDH group and were located very close in phylogenetic tree analysis [31]. In this study, the phylogenetic analysis of *OsGAPDHC* genes showed that *OsGAPDHC1* and *OsGAPDHC6* were located very closely, with values of 0.087 and 0.082, respectively (Supplemental Figure S1A). In addition, multiple alignment and gene structure analysis of the amino acid sequences of *OsGAPDHC1* and *OsGAPDHC6* showed that the NAD-binding domain and C-terminal domain were well conserved (Supplemental Figures S1B and S2A,B). qRT-PCR analysis was performed to determine the expression levels of *OsGAPDHC1* and *OsGAPDHC6* in the organs of rice plants (Figure 1). The expression levels of *OsGAPDHC1* and *OsGAPDHC6* were very high in seeds and lowest in leaves. In general, *OsGAPDCH1* showed higher expression levels than *OsGAPDHC6*, but the level of *OsGAPDHC6* was higher in seeds. Therefore, *OsGAPDHC1* and *OsGAPDHC6* had different expression levels compared to our previously reported organ-specific expression levels [31]. The expression levels of both genes were higher in the early germination stage compared to the reproductive stage (Figure 1).

### 3.2. Analysis of Expression Levels of OsGAPDHC1 and OsGAPDHC6 Under Abiotic Stress

**Figure 2:**
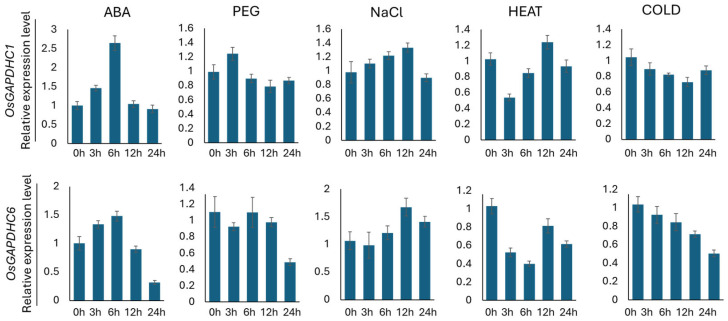
The analysis of the expression levels of *OsGAPHDC1* and *OsGAPHDC6* gene in seeds under abiotic stress (ABA, PEG, NaCl, heat, cold) conditions. The values 0 h, 3 h, 6 h, 12 h, and 24 h represent the treatment time for each abiotic stress. The 0 h samples of each abiotic stress condition were used as standards. Error bars represent the standard deviation calculated from three repetitions (mean ± SD, *n* = 3).

In this study, the expression levels of *OsGAPDHC1* and *OsGAPDHC6* genes were examined over time by treating various abiotic stresses (ABA, PEG, NaCl, heat, and cold) during rice seedlings. The expression levels of *OsGAPDHC1* and *OsGAPDHC6* were confirmed by qRT-PCR analysis, with sampling conducted for each stress treatment (Figure 2). As a result, the two gene expression patterns in the ABA- and NaCl-treated groups showed a constant pattern. In the ABA treatment group, the expression levels of *OsGAPDHC1* and *OsGAPDHC6* increased constantly from 0 h to 6 h but decreased sharply from 12 h onwards. In the NaCl treatment group, it was confirmed that the expression levels of *OsGAPDHC1* and *OsGAPDHC6* increased constantly from 0 h to 12 h but decreased to 24 h (Figure 2). In addition, the expression levels of both genes also differed over time in other abiotic stress treatments.

### 3.3. Generating Gene-Edited Mutants Using the CRISPR/Cas9 System

**Figure 3:**
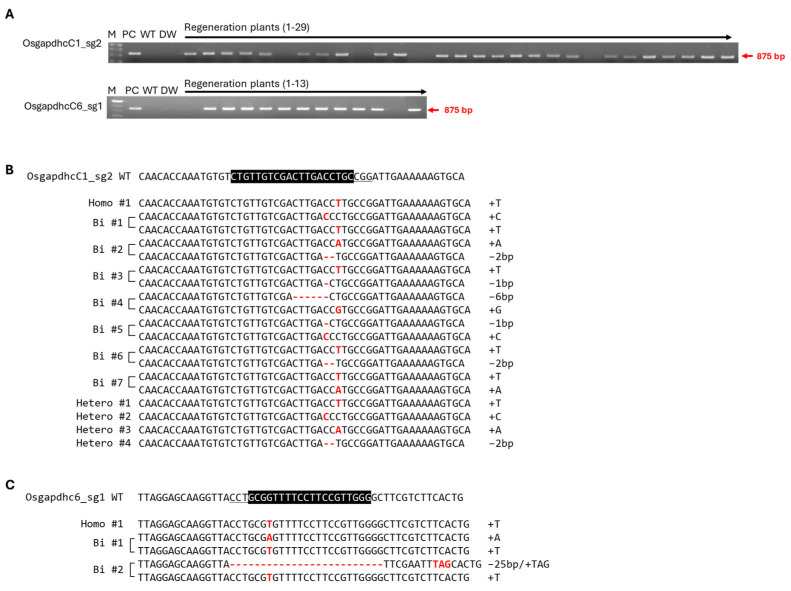
Selection of transgenic plants and gene-edited plants using deep-sequencing analysis. (**A**) Amplification of bar region and determination of T-DNA insertion by PCR analysis. M, 100 bp marker; PC, positive control; WT, wild type; DW, negative control. (**B**,**C**) Plants gene-edited using deep-sequencing analysis. The part highlighted in black represents sgRNA, and the underlined letters represent the PAM (NGG) region. Red letters indicate inserted bases, and dashes indicate deleted bases.

Because *OsGAPDHC1* and *OsGAPDHC6* responded to ABA and NaCl during seed germination under stress conditions, they were expected to be involved in the response mechanisms to both stresses. Therefore, in this study, the gene-edited plants were generated by using the CRISPR/Cas9 system to assess the role and function of the two genes (Supplementary Figure S2). *OsGAPDHC1* (Os02g07490) and *OsGAPDHC6* (Os06g45590) consisted of 14 exons and 13 intron regions. The sgRNA of the CRISPR/Cas9 system was designed in the 5th and 11th exon regions of *OsGAPDHC1* and the 2nd and 6th exon regions of *OsGAPDHC6*. Each sgRNA was designed by selecting the NAD-binding domain. The C-terminal domain was the target site (Supplementary Figure S2, Supplementary Table S1). The pBOsC vector, constructed by introducing sgRNA, was transferred into the rice genome by *Agrobacterium* cv *EHA105* (Supplementary Figure S2). PCR analysis was performed with primers of the bar gene region present in Ti-plasmid using the regenerated plants. As a result, transgenic plants containing Ti-plasmid were selected, and gene-edited plants were selected by deep-sequencing analysis (Figure 3, Supplementary Table S2). As a result, various base mutations of homo, bi-allelic, and hetero were generated, and homo and bi-allelic types of gene-edited individuals were selected for each gene. The homo #1 (T insertion) selected from *OsGAPDHC1*_sg2 was marked as *gapdhc* 1-1, the bi #2 (A insertion/2 bp deletion) was marked as *gapdhc* 1-2, the homo #1 (T insertion) selected from *OsGAPDHC6*_sg1 was marked as *gapdhc* 6-1, and the bi #1 (A/T insertion) was marked as *gapdhc* 6-2 (Figure 3B,C).

### 3.4. Characteristic of Derived Seeds from Gene-Edited Lines 

**Figure 4:**
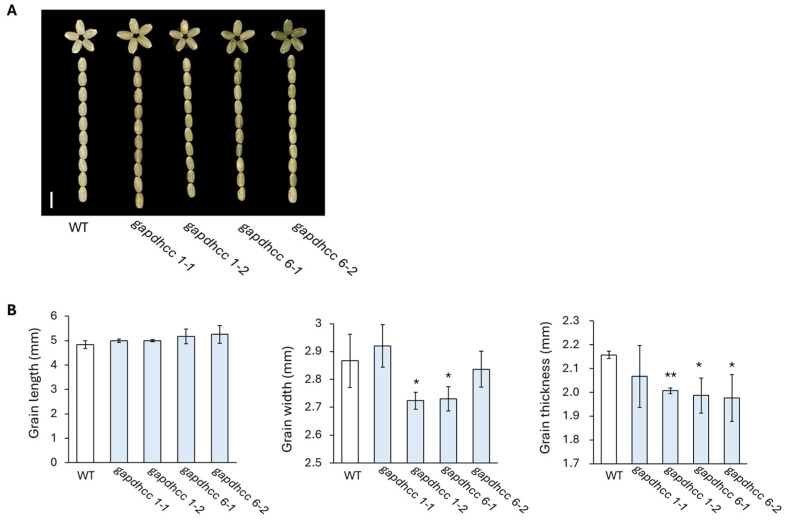
Phenotype analysis of seeds in T_1_ generation. (**A**) Seeds of WT and T_1_ mutants. Bar = 0.5 cm. (**B**) Measurements of seed length, width, and thickness of WT and T_1_ mutant lines. Error bars represent standard deviation in ten replicates (mean ± SD, *n* = 10). Asterisks indicate differences between WT and mutants (0.5 < * *p*, 0.05 < ** *p* < 0.5).

T_1_ seeds were harvested from four lines (*gapdhc* 1-1, *gapdhc* 1-2, *gapdhc 6*-1, and *gapdhc* 6-2) edited with the homo and bi-allelic selected in this experiment. In our experiment, the expression levels of *OsGAPDHC1* and *OsGAPDHC6* genes within the seeds were significantly different (Figure 1). Therefore, when examining the phenotypes of KO mutant seeds, we found that they showed large differences in grain length, grain width, and grain thickness in each KO line (Figure 4). Specifically, the thickness of the seeds tended to decrease in all lines, and the width of the seeds decreased, except for in the *gapdhc* 1-1 line (Figure 4B).

### 3.5. Response to Salt Stress in KO Lines Generated from GAPDHC6 Gene 

**Figure 5:**
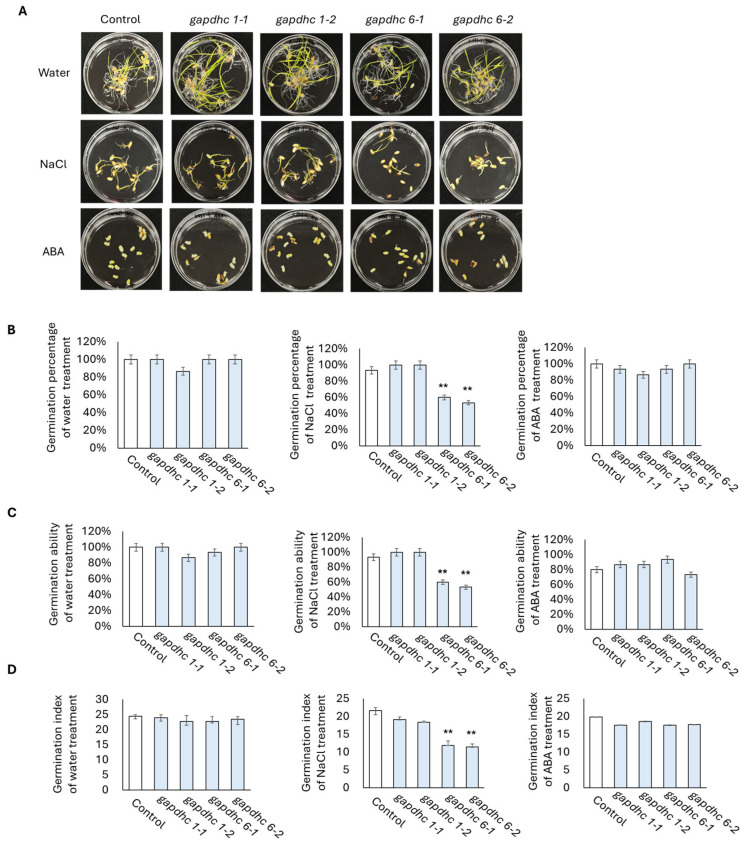
Germination experiments of KO mutant lines under water, salt, and ABA conditions. (**A**) Seeds of WT and T1 mutant lines were germinated for 10 days under conditions of water, NaCl (200 mM), and ABA (100 μM). (**B**) Germination rate. Measured on the 10th day after seed soaking. (**C**) Germination ability. The number of germinated seeds was measured on the 4th day of seed soaking. (**D**) Germination index. The number of germinated seeds per day was recorded. Error bars represent standard deviation in three replicates (mean ± SD, *n* = 3). Asterisks indicate differences between WT and mutants (0.05 < ** *p* < 0.5).

**Figure 6:**
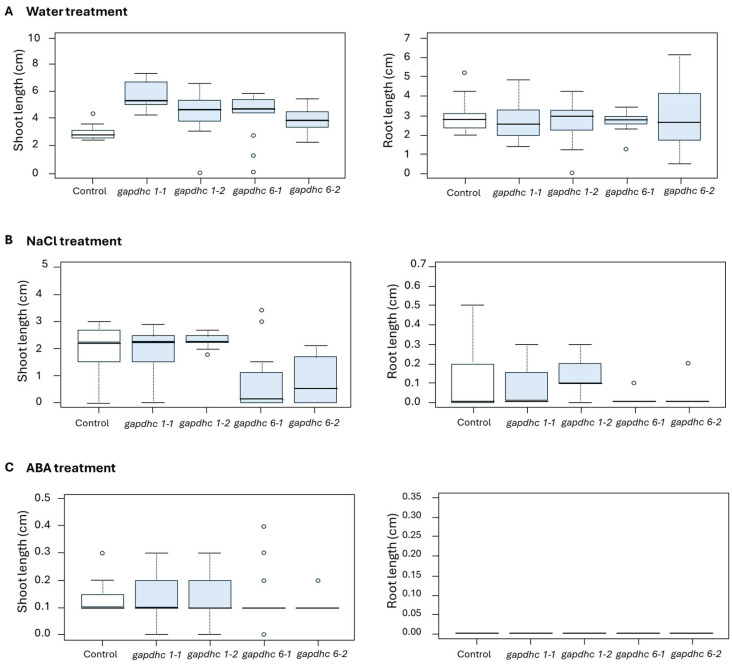
Measurement of shoot and root length growth of KO mutant lines under water, salt, and ABA conditions. (**A**) Measurement of shoot and root lengths of WT and KO mutant lines undergoing water treatment. (**B**) Measurement of shoot and root lengths of WT and KO mutant lines undergoing 200 mM NaCl treatment. (**C**) Measurement of shoot and root lengths of WT and KO mutant lines undergoing 100 μM ABA treatment. “°” represents a distributed individual value.

Seed germination experiments were conducted on each of the four selected KO lines using a solution containing water, 200 mM NaCl, and 100 μM ABA. The germination rate, germination power, and germination index were examined daily for 10 days from the start date, and finally, the shots and roots lengths were examined. The germination rate, germination ability, and germination index of all the lines examined in the water and ABA treatment showed almost similar results. However, in the NaCl treatment, the germination rate, germination ability, and germination index of the *gapdhc* 6-1 and *gapdhc* 6-2 lines were very low compared to the WT (Figure 5). In addition, in the shoot and root lengths of the *gapdhc* 6-1 and *gapdhc* 6-2 lines of the NaCl treatment, significant differences were confirmed compared to the control, *gapdhc* 1-1, and *gapdhc* 1-2 lines (Figure 6). Therefore, the *gapdhc* 6-1 and *gapdhc* 6-2 lines showed a sensitive response to salt.

### 3.6. Expression Level of Stress Response Related Genes in KO Mutant Lines

**Figure 7:**
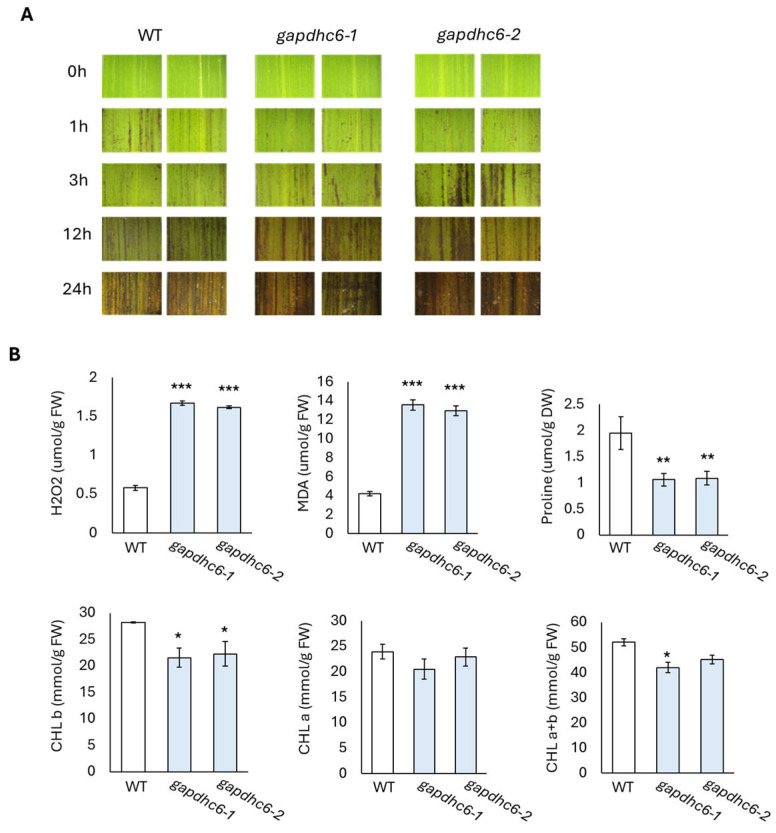
Analysis of ROS and antioxidant accumulation in leaves of WT and *gapdhc6* mutants. (**A**) Cell death analysis using DAB staining. (**B**) Evaluation of ROS and antioxidant contents. Error bars represent standard deviation in three replicates (mean ± SD, *n* = 3). Asterisks indicate differences between WT and mutants (0.5 < * *p*, 0.05 < ** *p* < 0.5, *** *p* < 0.05).

**Figure 8:**
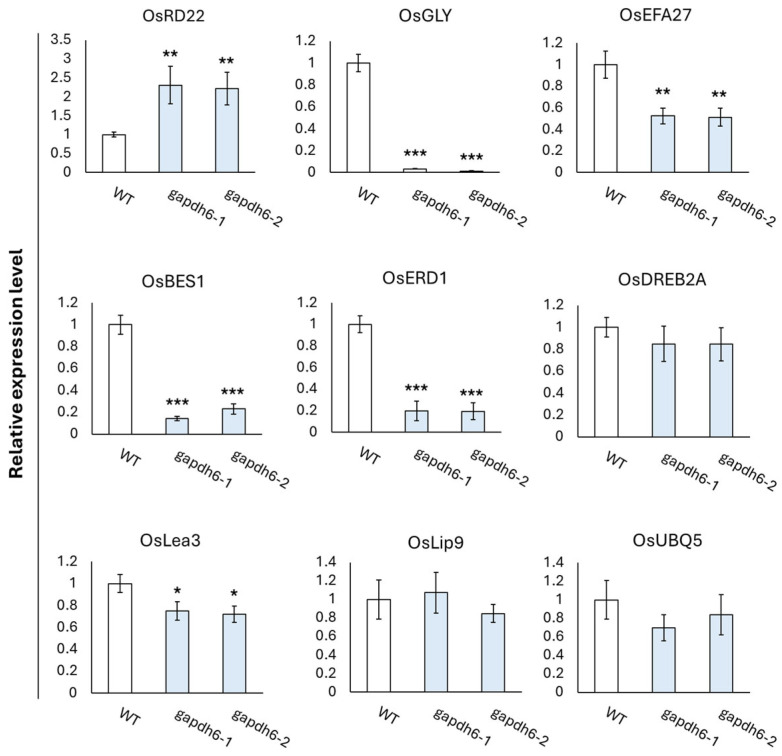
Relative expression levels of salt stress response genes in leaves of WT and *gapdhc6* mutants. Error bars represent standard deviation in three replicates (mean ± SD, *n* = 3). Asterisks indicate differences between WT and mutants (0.5 < * *p*, 0.05 < ** *p* < 0.5, *** *p* < 0.05).

In the germination experiment, the *gapdhc* 6-1, *gapdhc* 6-2, and WT plants were transferred tp pots and then the recovery process was carried out through water treatment for 15 days. The leaves of each seedling were sampled to examine the dynamics of stress reactants. The DAB staining of leaf disks of the *gapdhc* 6-1 and *gapdhc* 6-2 lines showed a marked difference at 3, 12, and 24 h compared to the WT. Therefore, the leaves of the *gapdhc* 6-1 and *gapdhc* 6-2 lines showed a sensitive reaction following NaCl treatment, considering that NaCl treatment easily led to cell death and a darker color was observed (Figure 7A). In addition, the ROS substance content in each leaf was measured, and the *gapdhc* 6-1 and *gapdhc* 6-2 lines accumulated very high levels of H_2_O_2_ and MDA compared to the WT (Figure 7B). The proline content of *gapdhc* 6-1 and *gapdhc* 6-2 lines was lower than that of WT, and the chlorophyll content was also slightly reduced. Therefore, we analyzed the expression levels of nine stress-responsive genes in *gapdhc* 6-1 and *gapdhc* 6-2 lines using qRT-PCR analysis. As a result, the gene expression levels of *OsGLY*, *OsEFA27*, *OsBES1*, *OsERD1*, and *OsLea3* were decreased in *gapdhc* 6-1 and *gapdhc* 6-2 lines compared to WT. On the other hand, the expression level of *OsRD22* was higher in the mutant lines than in WT (Figure 8).

## 4. Discussion

Rice is susceptible to various environmental stresses that significantly affect its productivity and quality. Among these stresses, salt stress is considered one of the major serious factors limiting crop production and plant growth in agriculture. In this study, we generated plants that were gene-edited with *OsGAPDHC1* and *OsGAPDHC6* using the CRISPR/Cas9 system, and selected KO lines were evaluated to assess responses to salt stress by examining seeds germination, gene expression levels, and ROS accumulation under salt stress. In the phylogenetic tree analysis, *OsGAPDHC1* and *OsGAPDHC6* were selected because they were very close to the cytosolic GAPDHC group, and their NAD^+^ binding domain and C-terminal domain were well conserved (Supplementary Figure S1). Expression levels of the *OsGAPDHC1* and *OsGAPDHC6* genes in rice were very high in seeds, but this differed from previously reported results [42]. In rice and *Arabidopsis*, GAPDHC was involved in seed development and energy metabolism, affecting seed size and oil content [42,43,44]. The change in the size of the T_1_ seed of the *gapdhc6* mutant generated through the CRISPR/Cas9 system was consistent with the results of these previous studies (Figure 4). In addition, these changes in seed size are thought to be related to the high expression levels of *OsGAPDHC1* and *OsGAPDHC6* in the seeds (Figure 1). Thus, in rice, GAPDH may be involved in plant growth and development, and it may also play an important role in stress responses [32,45,46]. Rice is known to be particularly vulnerable to salt stress at the seedling stage [47] and highly susceptible at the reproductive stage [48]. However, the early vegetative growth stage of rice from seed germination to first-stage tiller development is known to be relatively salt-resistant [42,49]. When the expression level of *OsGAPDHC* genes in WT rice seedlings was analyzed under various abiotic stresses, the expression of *OsGAPDHC1* and *OsGAPDHC6* appeared to be related to salt (Figure 2). Furthermore, in germination experiments, *gapdhc6* mutant lines were more sensitive to NaCl than WT, showing a lower germination rate, germination ability, and germination index, as well as reduced shoot and root length (Figure 5 and Figure 6). Salt stress can cause cell membrane damage, decreased CO_2_ uptake due to stomatal closure, decreased hydrolytic enzyme activity, and increased lipid peroxidation levels, which can stimulate the formation of ROS such as hydrogen peroxide [42,46]. The *osgapdhc6* mutant line accumulated about 3 times more hydrogen peroxide in leaves than the WT. In addition, the leaves of the *osgapdhc6* mutant line accumulated MDA 3.5 times more than the WT, and the leaf damage by DAB staining was also high (Figure 7). MDA is used as an indicator of oxidative damage that occurs during peroxidation due to stress-induced damage to lipid membranes [50]. Therefore, these results suggest that the *osgapdhc6* mutant line was more affected by lipid peroxidation than the WT, resulting in greater damage to cell membrane integrity. Proline is generally known to act as a physiologically compatible solute, and its presence increases as needed to maintain favorable osmotic potential between cells and the surrounding environment [51]. Proline that accumulates in plants under salt stress can contribute to root growth [52], but the proline concentration of the *osgapdhc6* mutant line was reduced by about half compared to the WT, and the root length was also the shortest (Figure 6B and Figure 7). Therefore, these results suggest that the knockout of *OsGAPDHC6* gene is sensitive to salt stress and can affect plant growth by causing oxidative stress. The *OsGAPDHC6* gene may interact with genes related to stress response to contribute to salt tolerance. In the *osgapdhc6* mutant lines, the relative expression levels of *OsGly*, *OsEFA27*, *OsBES1*, *OsERD1*, and *OsLea3* genes were significantly lower than those in the WT. The overexpression of *OsGly I* in rice enhanced tolerance to NaCl, ZnCl_2_, and mannitol [53], and overexpressed transgenic rice cultivars showed increased GLYI activity and enhanced salt stress tolerance, whereas the CRISPR/Cas9 knockout transgenic rice cultivars showed decreased GLYI activity and increased sensitivity to NaCl stress treatment [54]. EFA27 was identified as an ABA-responsive gene in rice seedlings [55], and in *Arabidopsis*, BES1 RNA interference (BES1-RNAi) lines were more sensitive to salt stress [47]. The overexpression of *BRI1-EMS-Suppressor 1* (*BES1*) in *Arabidopsis* resulted in lower MDA, higher proline content, enhanced antioxidant enzyme activities, and higher expression levels of salt-responsive genes under salt stress [56]. Transgenic *Arabidopsis* plants overexpressing the *BjERD4* gene exhibited enhanced tolerance to dehydration and salt stress, whereas the knockdown lines were more sensitive than wild-type plants under similar stress conditions [57]. The knockout of the *Lea3* gene significantly increased the sensitivity of cotton plants to salt and drought stress, which was due to the loss of its strong interaction with glyceraldehyde-3-phosphate dehydrogenase A [58]. The overexpression of *OsLEA3*-2 improved growth performance compared to the control under salt and osmotic stress conditions [59]. These results were consistent with our experimental results. However, the *OsRD22* gene showed a high expression level (Figure 8), which is inconsistent with the study result showing that the overexpression of the *RD22* gene contributes to salt tolerance in tobacco plants [60]. The overexpression of *OsDREB2A* results in significant tolerance to osmotic, salt, and dehydration stress under stress conditions [61], and T_2_ and T_3_ transgenic lines overexpressing *OsDREB2A* have improved survival rates under severe drought and salt stress conditions compared to non-transgenic rice plants or rice plants transformed with empty vector controls [62], but our results are similar to those of WT varieties. Nevertheless, the decrease in the expression levels of *OsGly*, *OsEFA27*, *OsBES1*, *OsERD1*, and *OsLea3* genes related to stress and response is suggested to be sufficient to determine the presence of salt-sensitivity. Therefore, the results of these experiments indicate that *OsGAPDHC6* can increase salt tolerance by participating in the expression of stress-responsive genes in the early growth stages, from seed germination to just before first-stage tiller development.

## Data Availability

The original contributions presented in the study are included in the article/Supplementary Materials, further inquiries can be directed to the corresponding authors.

## Supplementary Materials

The following supporting information can be downloaded at https://www.mdpi.com/article/10.3390/genes16040436/s1, Supplementary Figure S1: Phylogenetic analysis of OsGAPDHC and multiple sequence alignment of OsGAPDHC1 and OsGAPDHC6 amino acids. (A) Phylogenetic analysis of GAPDHC in rice. (B) Multiple sequence alignment of amino acid sequences encoding OsGAPDHC6 and OsGAPDHC1 proteins. Identical, conserve residues in all aligned sequences are indicated by asterisks (*); Supplementary Figure S2: Development of *OsGADPHC1* and *OsGAPDHC6* transgenic plants using the CRISPR/Cas9 system. (A) Schematic diagram of *OsGADPHCC1* gene map and sgRNA design. (B) Schematic diagram of *OsGAPDHCC6* gene map and sgRNA design. Red triangles and white dotted lines indicate the positions of sgRNA. (C) *OsGADPHCC1*::sgRNA transformation and production of regenerated rice plants using *Agrobacterium*-mediated transformation. (D) *OsGAPDHCC6*::sgRNA transformation and production of regenerated rice plants using *Agrobacterium*-mediated transformation; Supplementary Table S1: sgRNA designed in this study; Supplementary Table S2: Transgenic plant production and gene editing rates; Supplementary Table S3: The primers list used in this study.
