# Supplementary material for: A Knockout of the OsGAPDHC6 Gene Encoding a Cytosolic Glyceraldehyde-3-Phosphate Dehydrogenase Reacts Sensitively to Abiotic Stress in Rice
Source: Genes (Basel). 2025 Apr 6;16(4):436. doi: 10.3390/genes16040436 (PMC12027454; doi:10.3390/genes16040436)
Supplement: Supplementary file 1 [file genes-16-00436-s001.zip › genes-3551087-supplementary.pdf]

Supplementary Data

Knockout of *OsGAPDHC6* Gene Encoding a Cytosolic Glyceraldehyde-3-Phosphate Dehydrogenase Reacts Sensitively to Salt Stress in Rice

Jin Young Kim [1,†], Ye Ji Lee [1,†], Hye Mi Lee [1], Yoo Seob Jung [1], Jiyun Go [2], Hyo Ju Lee [1], Ki Sun Nam[1], Jong Hee Kim [1] Kwon Kyoo Kang [1,3,*] and Yu Jin Jung [1,3,*]

Contents

Supplementary Figure S1. Phylogenetic analysis of OsGAPDHC and multiple sequence alignment of OsGAPDHC1 and OsGAPDHC6 amino acids. **A,** Phylogenetic analysis of GAPDHC in rice. **B,** Multiple sequence alignment of amino acid sequences encoding OsGAPDHC6 and OsGAPDHC1 proteins. Identical, conserve residues in all aligned sequences are indicated by asterisks(*).

Supplementary Figure S2. Development of *OsGADPHCC1* and *OsGAPDHCC6* transgenic plants using the CRISPR/Cas9 system. **A,** Schematic diagram of *OsGADPHCC1* gene map and sgRNA design. **B,** Schematic diagram of *OsGAPDHCC6* gene map and sgRNA design. Red triangles and white dotted lines indicate the positions of sgRNA. **C,** *OsGADPHCC1*::sgRNA transformation and production of regenerated rice plants using *Agrobacterium*-mediated transformation. **D,** *OsGAPDHCC6*::sgRNA transformation and production of regenerated rice plants using *Agrobacterium*-mediated transformation.

Supplementary Table S1. sgRNA designed in this study.

Supplementary Table S2. Transgenic plant production and gene editing rates.

Supplementary Table S3. The primers list used in this study.

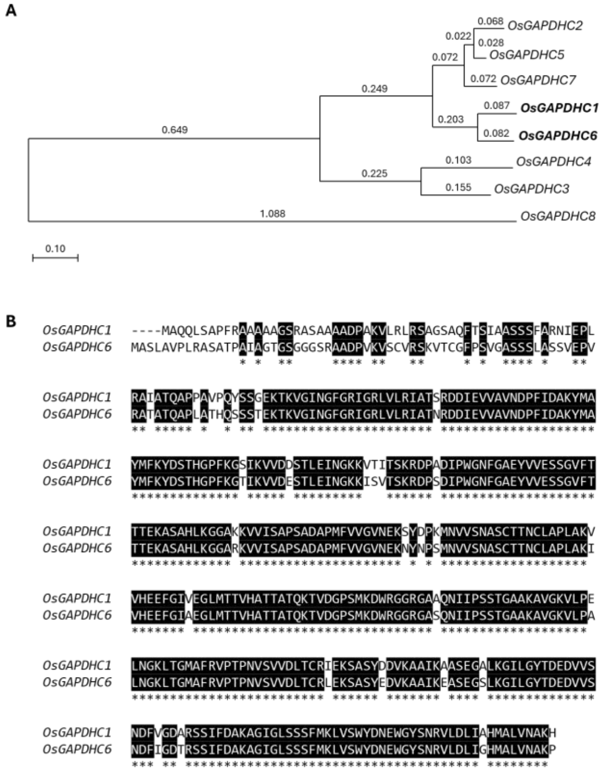

Supplementary Figure S1. Phylogenetic analysis of OsGAPDHC and multiple sequence alignment of OsGAPDHC1 and OsGAPDHC6 amino acids. **A,** Phylogenetic analysis of GAPDHC in rice. **B,** Multiple sequence alignment of amino acid sequences encoding OsGAPDHC6 and OsGAPDHC1 proteins. Identical, conserve residues in all aligned sequences are indicated by asterisks(*).

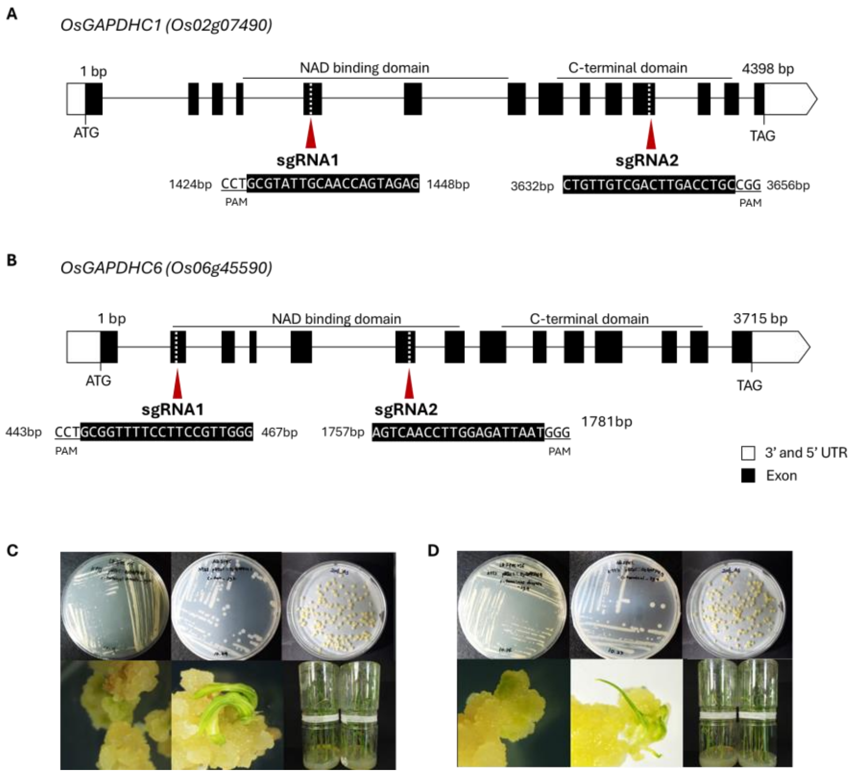

Supplementary Figure S2. Development of *OsGADPHCC1* and *OsGAPDHCC6* transgenic plants using the CRISPR/Cas9 system. **A,** Schematic diagram of *OsGADPHCC1* gene map and sgRNA design. **B,** Schematic diagram of *OsGAPDHCC6* gene map and sgRNA design. Red triangles and white dotted lines indicate the positions of sgRNA. **C,** *OsGAPDHCC1*::sgRNA transformation and production of regenerated rice plants using *Agrobacterium*-mediated transformation. **D,** *OsGAPDHCC6*::sgRNA transformation and production of regenerated rice plants using *Agrobacterium*-mediated transformation.

Supplementary Table S1. sgRNA designed in this study.

Gene Name	RGEN Target (5' to 3')	Direction	GC Contents (%, w/o PAM)	Out-of-frame Score	Mismatches			
					0	1	2	3
OsGAPDHC1 sg1	GCGTATTGCAACCAGTAGAGAGG	-	50	50	1	0	0	0
OsGAPDHC1 sg2	CTGTTGTCGACTTGACCTGCCGG	+	55	67.6	1	0	0	0
OsGAPDHC6 sg1	CCCAACGGAAGGAAAACCGCAGG	-	60	70.5	1	0	0	0
OsGAPDHC6 sg2	AGTCAACCTTGGAGATTAATGGG	+	35	65.1	1	0	0	0

Supplementary Table 2. Transgenic plant production and gene editing rates.

Gene Name	No. infected Calli	No. induced shoots	No. induced roots	No. Transgenic plants (%)	No. Edited plant (%)	Edited Genotypes		
						Homozygous	Bi-allelic	Heterozygous
OsGAPDHC1 sg1	80	13	11	10 (12.5%)	0	0	0	0
OsGAPDHC1sg2	80	62	43	43 (53.75%)	29 (67.4%)	4/29	12/29	13/29
OsGAPDHC6 sg1	80	44	20	19 (23.75%)	13 (68.4%)	1/13	12/13	0/13
OsGAPDHC6 sg2	80	4	0	0 (0%)	0	0	0	0

Supplementary Table S3. The primers list used in this study.

Primer name	Sequence (primer direction 5′-3′)	purpose
J67 pBOsC sgSEQ - FW	CAGCTTGGCTCTAGTCGACC	Vector construction
K20 RGEN scaaffold regoin RV	CGGTGCCACTTTTTCAAGTT	
OsGAPDHC1 sg1 up	ggcagGCGTATTGCAACCAGTAGAG	
OsGAPDHC1 sg1 down	aaacCTCTACTGGTTGCAATACGCc	
OsGAPDHC1 sg2 up	ggcagCTGTTGTCGACTTGACCTGC	
OsGAPDHC1 sg2 down	aaacGCAGGTCAAGTCGACAACAGc	
OsGAPDHC6 sg1 up	ggcagCCCAACGGAAGGAAAACCGC	
OsGAPDHC6 sg1 down	aaacGCGGTTTTCCTTCCGTTGGGc	
OsGAPDHC6 sg2 up	ggcagAGTCAACCTTGGAGATTAAT	
OsGAPDHC6 sg2 down	aaacATTAATCTCCAAGGTTGACTc	
T-DNA confirm-Nos ter Fw	TTGCGCGCTATATTTTGTTTT	T-DNA confirm
T-DNA confirm-Bar R Rv	CGTCAACCACTACATCGAGA	
OsGAPDHC1 sg2 1st F1	CAAGGATGGAGACTGCCAAT	
OsGAPDHC1 sg2 1st R1	GTGCATGACCGCAGTTTCTA	
OsGAPDHC1 sg2 2nd F1	ACACTCTTTCCCTACACGACGCTCTTCCGATCTGATTTCTGTTTTGGCTGACTG	
OsGAPDHC1 sg2 2nd R1	GTGACTGGAGTTCAGACGTGTGCTCTTCCGATCTATTGACCAAACAGAAACAGCA	
OsGAPDHC6 sg1 1st F1	TGTTGTTCGTATGGCGATGT	
OsGAPDHC6 sg2 1st R1	AACTTACGATGGGTGGCAAG	
OsGAPDHC6 sg2 2nd F1	ACACTCTTTCCCTACACGACGCTCTTCCGATCTTTCGCTTGATTTTCCGAGTT	
OsGAPDHC6 sg2 2nd R1	GTGACTGGAGTTCAGACGTGTGCTCTTCCGATCTCTGTGCTCTCGGTTGTCAAA	
OsACTIN FW	CAACACCCCTGCTATGTACG	qRT-PCR analysis
OsACTIN RV	ATCACCAGAGTCCAACACAA	

OsGAPDHC1 qRT-PCR Fw	TTGACCTGCCGGATTGAAAA	qRT-PCR analysis
OsGAPDHC1 qRT-PCR Rv	TGCATCACCAACGAGTCATT	
OsGAPDHC6 qRT-PCR Fw	GTCGACGGTCCTTCAATGAA	
OsGAPDHC6 qRT-PCR Rv	CCATACCAGTGAGTTTCCCG	
OsRD22 qRT-PCR Fw	AAGGCTGTGGATGAAACGGT	
OsRD22 qRT-PCR Rv	AATCATGATCGACTCGCCGG	
OsGLY qRT-PCR Fw	CCACATCTCCTTCCAATGCG	
OsGLY qRT-PCR Rv	TCTCGATCATGAAGCCGTCG	
OsEFA27 qRT-PCR Fw	TGTGGCTTTCTTCGACCTGG	
OsEFA27 qRT-PCR Rv	CAGCTTGGCAGAGTTGGGTA	
OsBES1 qRT-PCR Fw	ATACCGGAGTGCGACGAGTC	
OsBES1 qRT-PCR Rv	GGCCGTCCCTTCTATCTCCA	
OsERD1 qRT-PCR Fw	TTGCCTCATTGTGGTCAGGG	
OsERD1 qRT-PCR Rv	TGAGGCCAACACGTGATCTC	
OsDREB2A qRT-PCR Fw	GCCGGCCACTATACCTTCTG	
OsDREB2A qRT-PCR Rv	CGCTCCTGACAAACACGTTT	
OsLea3 qRT-PCR Fw	GAGAAGGTGGAGAAGGCGAC	
OsLea3 qRT-PCR Rv	GTGGTAAAGGTGTGCTTGCC	
OsLip9 qRT-PCR Fw	GATTGGTCAAGGCCTGGTCA	
OsLip9 qRT-PCR Rv	CAGAATGCCCAGCCCAAAAC	
OsUBQ5 qRT-PCR Fw	CCGGTATGCAGATCTTCGTG	
OsUBQ5 qRT-PCR Rv	GCTTCCCTGCGAAAATCAGC	
